# Layer-specific speckle tracking analysis of left ventricular systolic function and synchrony in maintenance hemodialysis patients

**DOI:** 10.1186/s12872-019-01324-z

**Published:** 2020-01-09

**Authors:** Chang Liu, Zi-Ning Yan, Li Fan, Jun Huang, Dan Shen, Xiang-Ting Song

**Affiliations:** Department of Echocardiography, The Affiliated Changzhou No.2 People’s Hospital of Nanjing Medical University, Changzhou, 213003 China

**Keywords:** Echocardiography, Maintenance hemodialysis, Ventricular function, Synchronicity, Layer-specific strain

## Abstract

**Background:**

This study investigated the value of layer-specific strain analysis by two-dimensional speckle tracking echocardiography (2D-STE) for evaluating left ventricular (LV) systolic function and synchrony in maintenance hemodialysis (MHD) patients.

**Methods:**

A total of 34 MHD patients and 35 healthy controls were enrolled in this study. Dynamic images were collected at the LV apical long-axis, the four- and two- chamber, and the LV short-axis views at the basal, middle, and apical segments. The layer-specific speckle tracking (LST) technique was used to analyze the longitudinal strain (LS) and circumferential strain (CS) of LV sub-endocardium, mid-myocardium, sub-epicardium, global longitudinal strain (GLS), global circumferential strain (GCS), the LV 17 segment time to peak LS (TTP), and the peak strain dispersion (PSD). The differences in these parameters were compared between control and MHD groups, and the correlation between PSD and each LS parameter was examined. The receiver operator characteristic (ROC) curve was used to evaluate the efficacy of three myocardial layer LS and CS in the assessment of LV systolic dysfunction in MHD patients.

**Results:**

MHD patients had comparable left ventricular ejection fraction (LVEF), but significantly smaller LV GLS, GCS, and three-layer LS and CS compared to the control group. The three myocardial layer LS of the basal segment, middle segment, and apex segment was significantly reduced in the MHD patients compared to the normal subjects, while the three myocardial layer CS of the basal segment, middle segment, and apex segment was significantly reduced in the MHD patients compared to the normal subjects, except for the sub-endocardium of the middle and apex segment. MHD patients had significantly higher TTP of LV 17 segments and PSD compared to controls, and had delayed peak time in most segments. In addition, PSD of MHD patients was positively correlated with sub-endocardial and mid-myocardial LS and GLS, but not with sub-epicardial LS. The area under the curves (AUCs) of sub-endocardial, mid-myocardial, and sub-epicardial LS in MHD patients were 0.894, 0.852, and 0.870, respectively; the AUCs of sub-epicardial, mid-myocardial, and sub-endocardial CS were 0.852, 0.837, and 0.669, respectively.

**Conclusions:**

LST may detect early changes of all three-layer LS and CS and PSD in MHD patients, and is therefore a valuable tool to diagnose LV systolic dysfunction in MHD patients.

## Background

End stage renal disease (ESRD) is the final manifestation of chronic renal insufficiency. Hemodialysis, which removes blood metabolites, reduces sodium and water retention, and maintains electrolytes and acid-base balance through solute exchange, is the mainstay treatment for ESRD patients who cannot undergo kidney transplantation [1]. Long-term hemodialysis (i.e. maintenance hemodialysis, MHD) has been proven to be effective in reducing clinical symptoms and improving the quality of life of ESRD patients. However, despite the advances in hemodialysis technology, the mortality and morbidity of ESRD patients on MHD remains high and the quality of life of these patients is poor [2]. Cardiovascular disease is a major complication of ESRD that is caused by metabolic and hemodynamic changes due to ESRD and is associated with high mortality [3, 4], accounting for approximately 50% of hemodialysis patient deaths [5]. Left ventricular (LV) dysfunction is a critical indicator of cardiac dysfunction and an early clinical manifestation of LV hypertrophy. Thus, LV dysfunction holds important prognostic value for mortality of ESRD patients on MHD [6, 7]. There is a significant need to employ effective screening methods to identify early changes in cardiac function in ESRD patients on MHD.

Two-dimensional speckle tracking echocardiography (2D-STE), which can non-invasively, semi-automatically, and quantitatively analyze ventricular myocardial strain function, has been widely used in the clinic to evaluate cardiac function [8, 9]. Compared with traditional echocardiography, 2D-STE technology is faster, more accurate, and angle-independent; it can assess myocardial function through longitudinal, circumferential, radial, and torsional motions [10], and thus can examine the global and local left ventricular function with high sensitivity. The layer-specific speckle tracking (LST) technology, derived from 2D-STE, can analyze strain parameters of LV function in three muscular layers – the sub-endocardium, mid-myocardium, and sub-epicardium [11]. Therefore, LST allows early detection of subtle ventricular systolic dysfunction [11–13]. Previous studies used the LST to assess global longitudinal, circumferential, and radial myocardial strains of dialysis patients [14–16], but few studies have been performed to measure longitudinal and circumferential strains of the LV three-layer myocardium. In addition, LV systolic synchrony is a sensitive parameter that may reflect early cardiac dysfunction. A normal heart has a well-synchronized LV systolic function, and dys-synchrony is observed in stress, such as in response to pharmacological stimulation [17]. However, using LST to evaluate LV systolic synchrony in MHD patients has been rarely reported.

In the present study, we used LST to evaluate the longitudinal and circumferential strains of the three-layer LV muscles in MHD patients and to study LV systolic synchrony, with the aim of evaluating the value of LST in detecting cardiac dysfunction at an early stage of MHD.

## Methods

### Ethics statement

This study protocol was approved by the Ethics Committee of Changzhou Second People’s Hospital affiliated to Nanjing Medical University, and all participants signed consent.

### Participant selection

A total of 34 ESRD patients on MHD who were admitted to the Changzhou Second People’s Hospital affiliated with Nanjing Medical University between September 2018 and June 2019 and 35 sex- and age-matched healthy individuals (the control group) were registered in this study. Patients who had all of the following were included in this study: 1) kidney disease as the primary disease; 2) LV ejection fraction (LVEF) ≥ 50%; 3) all MHD patients were treated with hemodialysis through forearm arteriovenous anastomosis; 4) hemodialysis was performed three times per week, 4 h each time; 5) hemodialysis lasted 10–36 months; and 6) each patient was weighed before and after hemodialysis, and the post-dialysis weight was equal to the ideal dry weight of each patient (clinically determined), and the difference in weight before and after dialysis was equal to the total volume of hemodialysis removed. All patients underwent image acquisition and measurement within 30 min after hemodialysis. Patients who had one of the following diseases were excluded from this study: congenital heart disease, valvular heart disease, coronary artery disease, cardiomyopathy and other heart diseases, arrhythmia, and a history of pulmonary hypertension. Control subjects had normal physical examinations, electrocardiograms, x-ray examinations, and echocardiography results, and did not have any of the following diseases: hypertension, diabetes, heart disease, or abnormal liver and kidney function.

### Data collection

The demographic and clinical data of all participants were obtained from interviews and the hospital database.

### Conventional 2D Doppler echocardiography

Thirty-four MHD patients and 35 control subjects underwent conventional 2D Doppler echocardiography (Vivid E9, GE). The M5S probe was used at a frequency of 2–4.5 MHz, and M-mode was used to measure the left ventricular internal diameter at end-diastole (LVIDD), left ventricular internal diameter at end-systole (LVIDS), interventricular septal thickness at end-diastole (IVST), left ventricular posterior wall thickness at end-diastole (LVPWT), left atrial diameter (LAD), and LVEF. Each parameter was measured three times and the average was used to determine the left ventricular mass index (LVMI) using the following formula: LVMI = left ventricular mass / body surface area; left ventricular mass (LVM) = 0.8 × 1.04 x [(LVIDD + LVPWT + IVST)^3^-LVIDD^3^] + 0.6; body surface area (BSA) = 0.0061 x height + 0.0128 x body weight - 0.1529.

Subjects were situated in the left lateral position, connected to the electrocardiogram, and requested to hold their breath to ensure image quality. The instrument was adjusted to clearly display the endocardium, with the image frame rate of 60–90 frames/s. The following images were collected during three consecutive cardiac cycles: LV apical long-axis, two- and four-chamber, and LV short-axis views of the basal, middle, and apical segments. All images were stored in the hard drive.

### Data analysis of LV systolic function

Dynamic images of the basal, middle, and apical segments obtained at the LV short-axis view, and dynamic images of the long-axis, two- and four- chamber views obtained at the LV apical view were imported into the 2D speckle tracking analysis software (2D-Strain, EchoPac. PC version 201, GE Healthcare, Horten, Norway) for analysis. Briefly, the LV endocardium in the apical long-axis chamber view was first delineated, followed by the automatic depiction of the region of interest (ROI), including the LV sub-endocardial, mid-myocardium, and sub-epicardial. Then, the curves of the endocardium and epicardium were manually adjusted to match the LV wall. The system automatically selected the aortic valve closing time point according to the ECG, and automatically generated the strain curves and values of each segment.

Using a similar method, the apical four- and two-chamber heart images were analyzed to obtain the sub-endocardial, mid-myocardial, and sub-epicardial longitudinal strain (LS) of LV 17 segments and their corresponding curves, as well as the bull’s eye diagram and to calculate the LV three-layer LS. The LV global longitudinal strain (GLS) is equivalent to the mean LS of the three layers of myocardium. The system also automatically generated LV 17 segment time to peak LS (TTP) and peak strain dispersion (PSD). Three dynamic images of the LV short axis were traced clockwise from the anterior septum, and the software automatically generated ROI including sub-endocardial, mid-myocardial, and sub-epicardial myocardium. If automatic tracking did not yield satisfactory outcomes, manual adjustment was then performed. The system automatically generated the myocardial circumferential strain (CS) of 17 segments and corresponding curves, and calculated the LV three-layer myocardial CS, the CS of the basal, middle, and apical segments. The LV global circumferential strain (GCS) is equivalent to the mean CS of the three layers of myocardium.

### Evaluation of intra-observer and inter-observer variability

Intra-observer and inter-observer variability were examined in this study. We randomly selected 20 subjects from control and MHD groups for this evaluation. The intra-observer differences were compared between the two observations made by the same observer at 1 week. The inter-differences between observers were compared between two independent observers who were blinded to the grouping. The intra-observer and inter-observer variabilities were evaluated using the intra-class correlation coefficients (ICCs).

### Statistical analysis

All statistical analyses were performed using SPSS 22.0 software (IBM SPSS, Statistics, Chicago, IL, USA). Measurement data are expressed as mean ± standard deviation (SD), and significance was set at the level of *P* < 0.05. The Kolmogorov-Smirnov’s test was used to evaluate data normality. The independent sample T test was used to compare normally distributed data, while the non-parametric Mann-Whitney test was used to compare non-normal data. Pearson correlation analysis was used to examine the correlation between normally distributed variables, while Spearman correlation analysis was used for non-normal variables. The LS and CS values ​​of the myocardium in each layer of the control subjects were defined as normal, and the LS and CS values ​​of the myocardium in each layer of MHD patients were defined as abnormal. Sub-endocardial, mid-myocardial, and sub-epicardial LS and CS in MHD patients were analyzed using the receiver operating characteristic curve (ROC). The Yoden index was used to calculate the critical point of each strain value, specificity, and sensitivity. The criteria for ICCs were: “excellent” if ≥0.80, “good” if 0.61–0.79, “moderate” if 0.41–0.60, and “poor” ICC ≤ 0.40.

## Results

### Comparison of demographic and clinical characteristics between control and MHD groups

We first compared demographic and clinical data between control and MHD groups. As shown in Table 1, MHD patients had significantly higher SBP, DBP, and creatinine (*P* < 0.05) compared to the control group, but there were no significant differences with regard to age, gender, heart rate, and body mass index (BMI) between these two groups (*P* > 0.05). The primary cause of ESRD was glomerulonephritis (55.8%), followed by diabetic nephropathy (17.6%), hypertensive nephrosclerosis (14.7%), and polycystic kidney (11.8%) (Table 1).

**Table 1:** Comparison of demographic and clinical characteristics between control and MHD groups

	MHD (*n* = 34)	Control (*n* = 35)	*P*-value
Age (years)	52 ± 9	49 ± 10	0.327
Male gender (%)	52.9	48.6	
HD time (months)	23(10–36)		
Heart rate (beats/min)	76.79 ± 9.63	72.34 ± 10.50	0.071
BMI	22.18 ± 2.11	21.29 ± 2.14	0.086
Remove volume (kg)	1.82 ± 0.93		
Dry weight (kg)	52.07 ± 8.95		
SBP (mmHg)	144.65 ± 17.00	121.40 ± 6.49	**< 0.001**
DBP (mmHg)	88.82 ± 11.17	75.91 ± 6.29	**< 0.001**
Creatinine (umol/L)	855.41 ± 191.36	55.86 ± 9.92	**< 0.001**
Cause of ESRD			
Glomerulonephritis (cases)	19 (55.8%)		
Diabetic Nephropathy (cases)	6 (17.6%)		
Hypertensive nephrosclerosis (cases)	5 (14.7%)		
Polycystic kidney (cases)	4 (11.8%)		

Data are expressed as mean ± SD. Bold number: *P* < 0.05

*HD* hemodialysis; *BMI* body mass index; *BP* systolic blood pressure; *DBP* diastolic blood pressure; Creatinine: serum creatinine; *ESRD* end-stage renal disease

### Comparison of traditional echocardiography parameters between control and MHD groups

We next compared traditional echocardiography parameters between control and MHD groups. As shown in Table 2, LVEF was comparable between the groups (*P* > 0.05), but MHD patients had significantly higher LVIDD, LVIDS, IVST, LVPWT, LAD, LVMI (*P* < 0.01) compared to the control subjects. These observations suggest that MHD patients had impaired cardiac function.

**Table 2:** Comparison of traditional echocardiography parameters between control and MHD groups

	MHD	Normal	P-value
LVIDD (mm)	52.56 ± 6.13	46.06 ± 3.07	**< 0.001**
LVIDS (mm)	36.65 ± 5.55	31.11 ± 2.84	**< 0.001**
IVST (mm)	11.56 ± 1.62	8.86 ± 1.38	**< 0.001**
LVPWT (mm)	11.24 ± 1.50	8.14 ± 1.38	**< 0.001**
LAD (mm)	42.38 ± 5.29	34.86 ± 3.12	**< 0.001**
LVEF (%)	58.24 ± 5.16	60.11 ± 4.00	0.095
LVMI (g/m^2^)	56.59 ± 9.11	31.60 ± 3.82	**< 0.001**

Data are expressed as mean ± SD. Bold number: *P* < 0.05

*LVIDD* left ventricular internal diameter at end-diastole; *LVIDS* left ventricular internal diameter at end-systole; *IVST* interventricular septal thickness at end-diastole; *LVPWT* left ventricular posterior wall thickness at end-diastole; *LAD* left atrial diameter; *LVEF* left ventricular ejection fraction; *LVMI* left ventricular mass index

### Comparison of layer-specific strain parameters between control and MHD groups

**Fig. 1:**
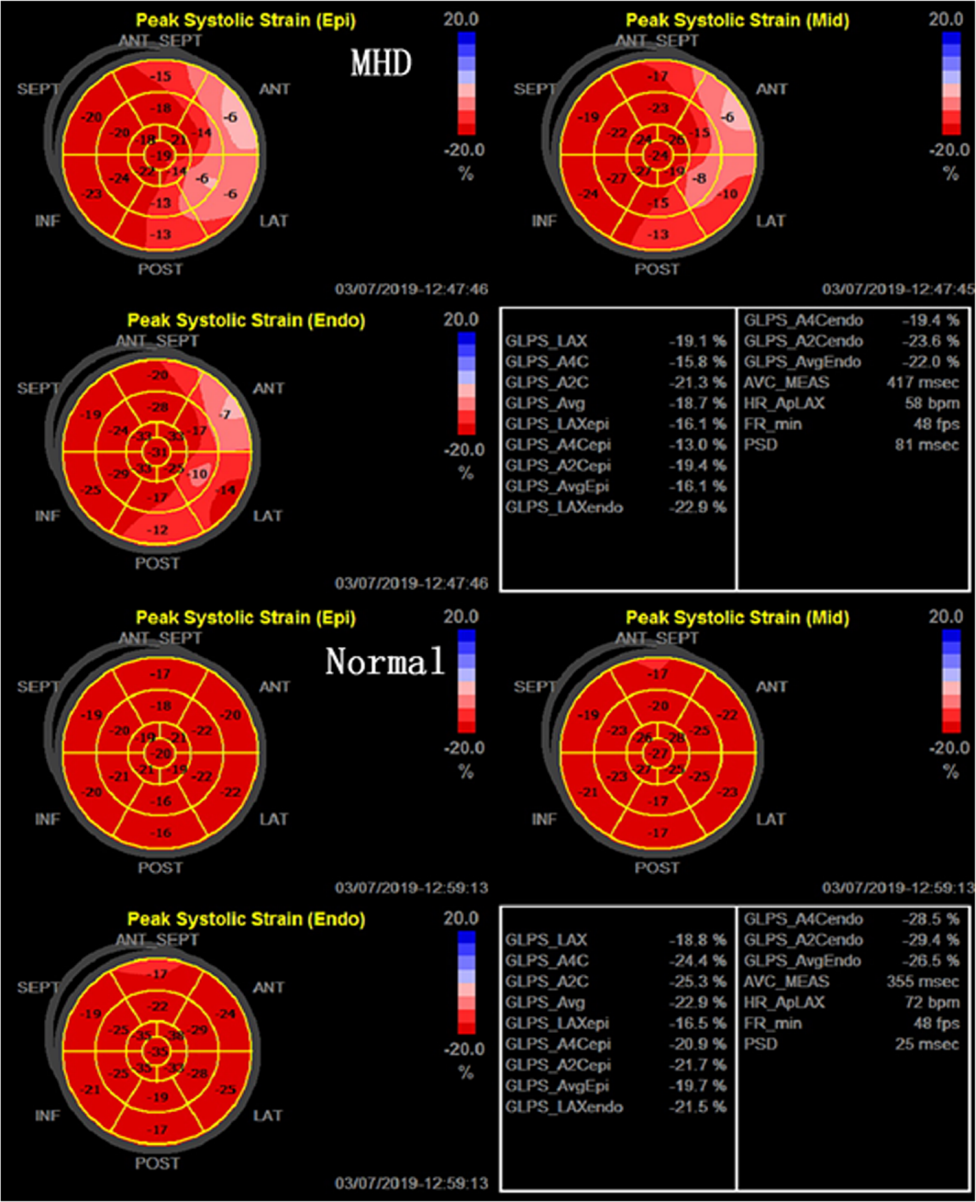
Bull’s eyes of the peak LS of the sub-endocardial, mid-myocardial, and sub-epicardial layers in MHD patients and normal subjects

**Fig. 2:**
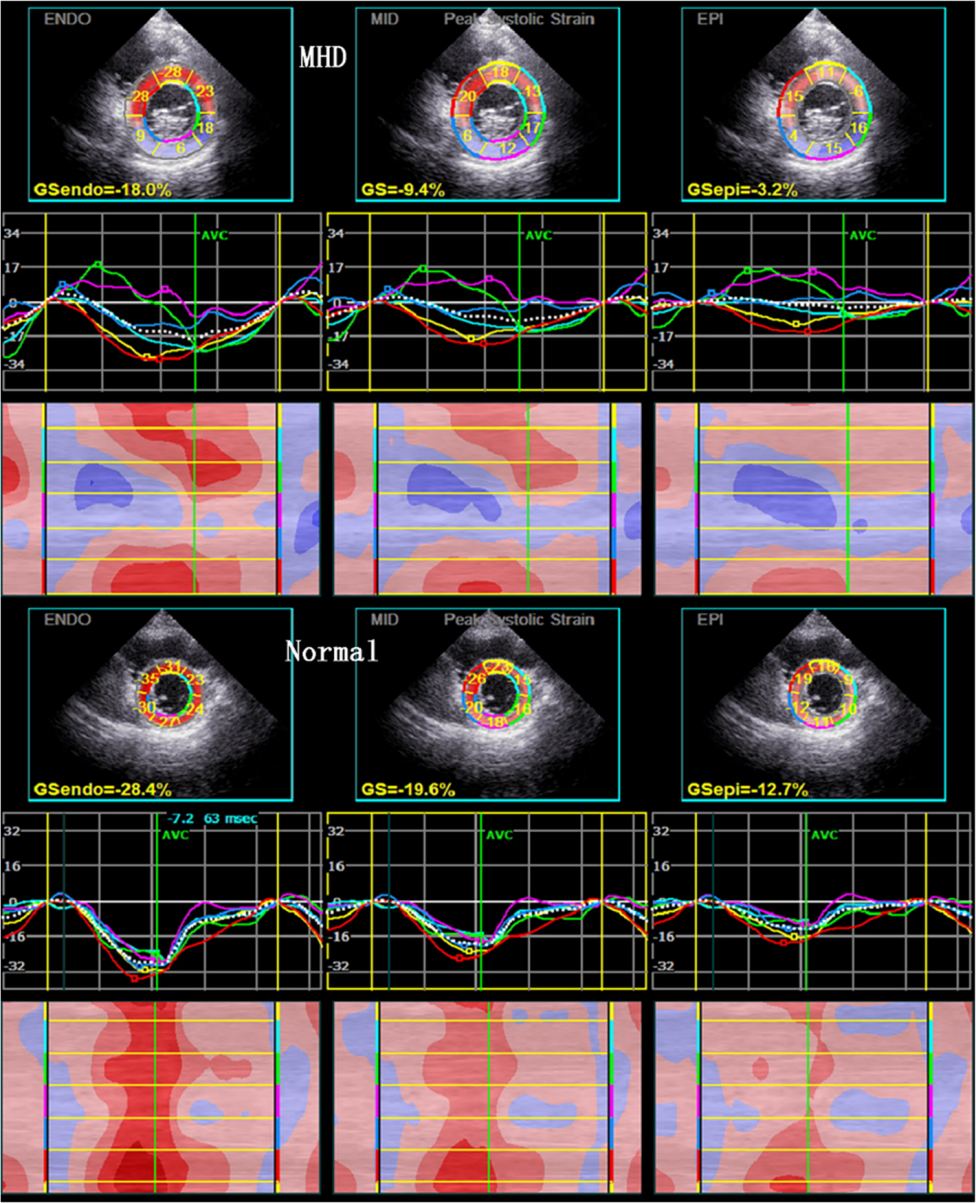
Peak CS of the sub-endocardial, mid-myocardial, and sub-epicardial layers of LV in MHD patients and normal subjects

In both groups, LS and CS of the LV three-layer myocardium were consistent with the following pattern: sub-endocardial > mid-myocardium > sub-epicardial. Compared with the control group, the MHD group had significantly lower LV GLS, GCS, and three-layer myocardial LS and CS (*P* < 0.01) (Table 3). MHD patients also had significantly lower LS of the LV three-layer at the basal, middle, and apical segments (*P* < 0.01) (Table 4, Fig. 1). In addition, with the exception of the sub-endocardium at the apical and middle segments, MHD patients had significantly lower CS of other LV layers compared to the control group (*P* < 0.05 or *P* < 0.01) (Table 5, Fig. 2).

**Table 3:** Comparison of global and layer-specific LS and CS between control and MHD groups

Varible	Longitudinal strain (%)				Circumferential strain(%)			
	Sub-endocardial	Mid-myocardial	Sub-epicardial	Global	Sub-endocardial	Mid-myocardial	Subepicardial	Global
MHD	−17.04 ± 4.09	−14.76 ± 4.11	−13.50 ± 2.99	−15.07 ± 3.46	−24.70 ± 4.68	−15.33 ± 2.44	−9.20 ± 1.67	−16.41 ± 2.59
Normal	−23.41 ± 3.58	−20.18 ± 3.25	−18.07 ± 2.75	−20.55 ± 3.16	−27.40 ± 3.51	−18.94 ± 2.74	−12.36 ± 2.52	−19.57 ± 2.75
*P*-value	**< 0.001**	**< 0.001**	**< 0.001**	**< 0.001**	**0.008**	**< 0.001**	**< 0.001**	**< 0.001**

Data are expressed as mean ± SD. Bold number: *P* < 0.05

**Table 4:** Comparison of horizontal layer-specific longitudinal strain between control and MHD groups

Segmental LV wall	Longitudinal strain (%)								
	Endocardial			Middle			Epicardial		
	MHD	Normal	P-value	MHD	Normal	P-value	MHD	Normal	P-value
basal segment	−12.47 ± 4.23	−19.57 ± 2.92	**< 0.001**	− 12.15 ± 4.32	−18.66 ± 2.58	**< 0.001**	−12.15 ± 3.53	− 18.06 ± 2.75	**< 0.001**
middle segment	−16.56 ± 4.42	−22.40 ± 3.42	**< 0.001**	− 14.82 ± 3.93	− 19.83 ± 3.28	**< 0.001**	− 14.38 ± 3.14	−18.37 ± 2.61	**< 0.001**
apex segment	−22.09 ± 5.37	−28.26 ± 5.26	**< 0.001**	−17.32 ± 5.26	− 22.06 ± 4.54	**< 0.001**	−13.65 ± 4.12	− 17.77 ± 3.43	**< 0.001**

Data are expressed as mean ± SD. Bold number: *P* < 0.05

**Table 5:** Comparison of horizontal layer-specific circumferential strain parameters between control and MHD patients

Segmental LV wall	Circumferential strain(%)								
	Sub-endocardial			Mid-myocardial			sub-epicardial		
	MHD	Normal	*P*-value	MHD	Normal	*P*-value	MHD	Normal	*P*-value
basal segment	−23.88 ± 5.99	− 27.46 ± 5.37	**0.011**	−15.15 ± 3.60	−19.09 ± 4.18	**< 0.001**	−9.47 ± 2.62	−13.11 ± 3.64	**< 0.001**
middle segment	−23.79 ± 5.40	−26.00 ± 3.80	0.053	−14.44 ± 3.52	− 17.97 ± 3.26	**< 0.001**	−8.56 ± 3.15	−12.43 ± 3.17	**< 0.001**
apex segment	−26.41 ± 7.23	−28.74 ± 5.25	0.131	−16.41 ± 3.49	−19.77 ± 4.03	**< 0.001**	−9.56 ± 3.41	−11.54 ± 3.39	**0.018**

Data are expressed as mean ± SD. Bold number: *P* < 0.05

### Comparison of LV synchronous parameters between control and MHD groups

**Fig. 3:**
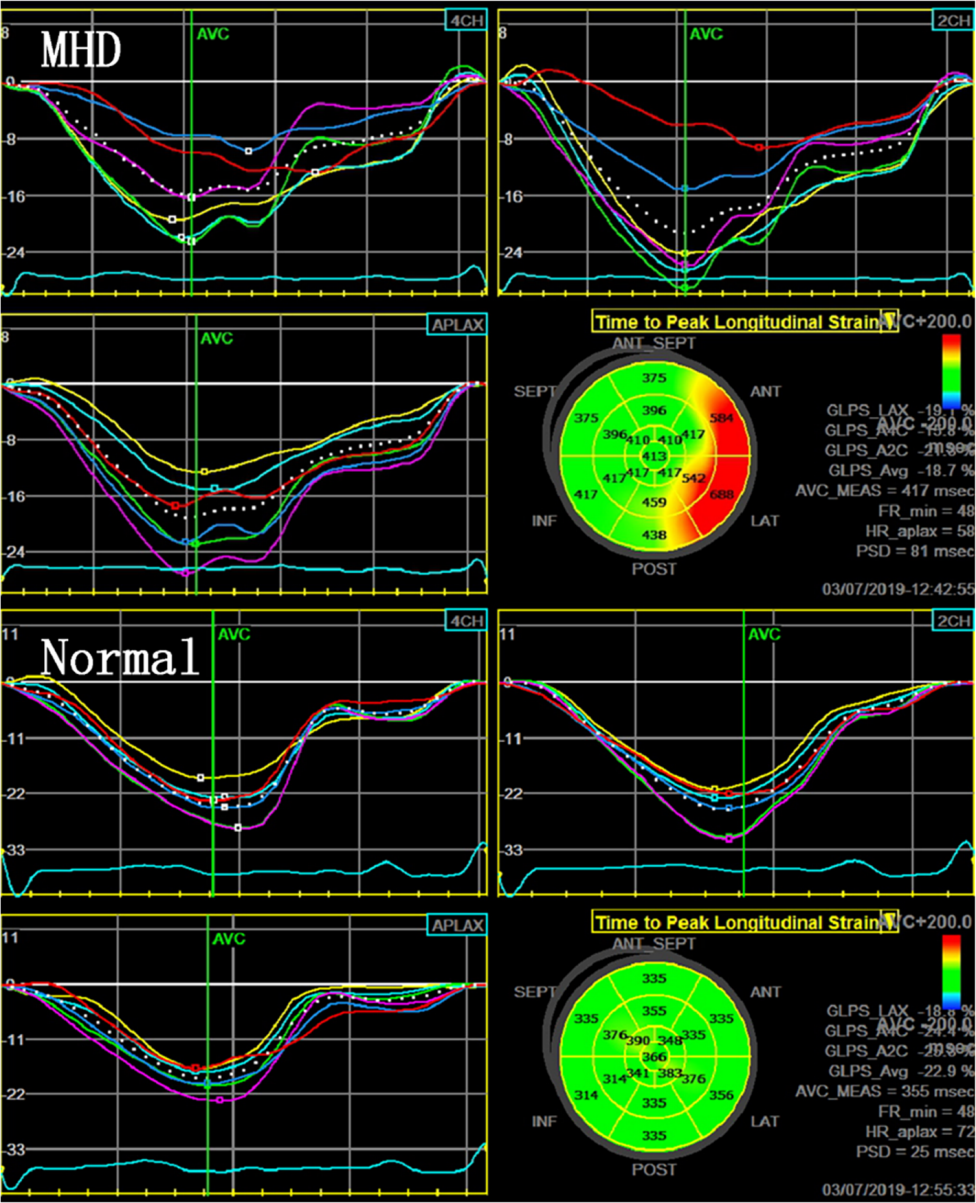
Bull’s eyes of the time to peak LS for the multiple LV segments and their PSD between MHD patients and normal subjects

In the control group, the color of the TTP bull’s eye was uniformly green, while the color of the bull’s eye in the MHD group was disordered, showing a mix of yellow and red, indicating that LV contraction was poorly synchronized (Fig. 3). The TTP of the LV 17 segments of MHD patients was significantly higher compared to the control group, and the peak time delay was observed in the majority of these segments (Table 6). In addition, MHD patients had significantly higher PSD compared to the control group (*P* < 0.01, Table 7). Taken together, these findings suggest that MHD patients had poorly synchronized LV systolic contraction, a sign of cardiac dysfunction.

**Table 6:** Comparison of time to peak longitudinal strain of LV 17 segments between control and MHD groups

variable	TTP (ms)		
	MHD	Normal	*P*-value
bas ANT-SEPT	415 ± 93	375 ± 55	**0.032**
bas ANT	424 ± 85	375 ± 53	**0.006**
bas LAT	451 ± 73	383 ± 57	**< 0.001**
bas POST	405 ± 54	379 ± 51	**0.045**
bas INF	394 ± 56	367 ± 33	**0.017**
bas SEPT	389 ± 71	376 ± 52	0.669
mid ANT-SEPT	375 ± 56	363 ± 42	0.499
mid ANT	403 ± 80	360 ± 39	**0.006**
mid LAT	431 ± 67	366 ± 38	**< 0.001**
mid POST	401 ± 51	366 ± 41	**0.002**
mid INF	381 ± 48	360 ± 30	**0.028**
mid SEPT	368 ± 52	360 ± 33	0.094
ap ANT	389 ± 60	365 ± 33	**0.044**
ap LAT	385 ± 59	369 ± 33	0.151
ap INF	397 ± 61	358 ± 28	**0.001**
ap SEPT	370 ± 46	362 ± 33	0.372
ap AP	383 ± 46	364 ± 28	**0.047**

Data are expressed as mean ± SD. Bold number: *P* < 0.05

*Bas* basal segment; mid: middle segment; *ap* apex segment; *ANT-SEPT* anterior septum wall; *ANT* anterior wall; *LAT* lateral wall; *POST* posterior wall;

*INF* inferior wall; *SEPT* septum wall; *TTP* time to peak longitudinal strain

**Table 7:** Comparison of LV PSD between control and MHD groups

Group	n	PSD
MHD	34	57.26 ± 18.18
Normal	35	29.97 ± 8.31
*P*-value		**< 0.001**

Data are expressed as mean ± SD. PSD: peak strain dispersion. Bold number: *P* < 0.05

### Correlation between PSD and GLS with three-layer LV myocardial LS

**Fig. 4:**
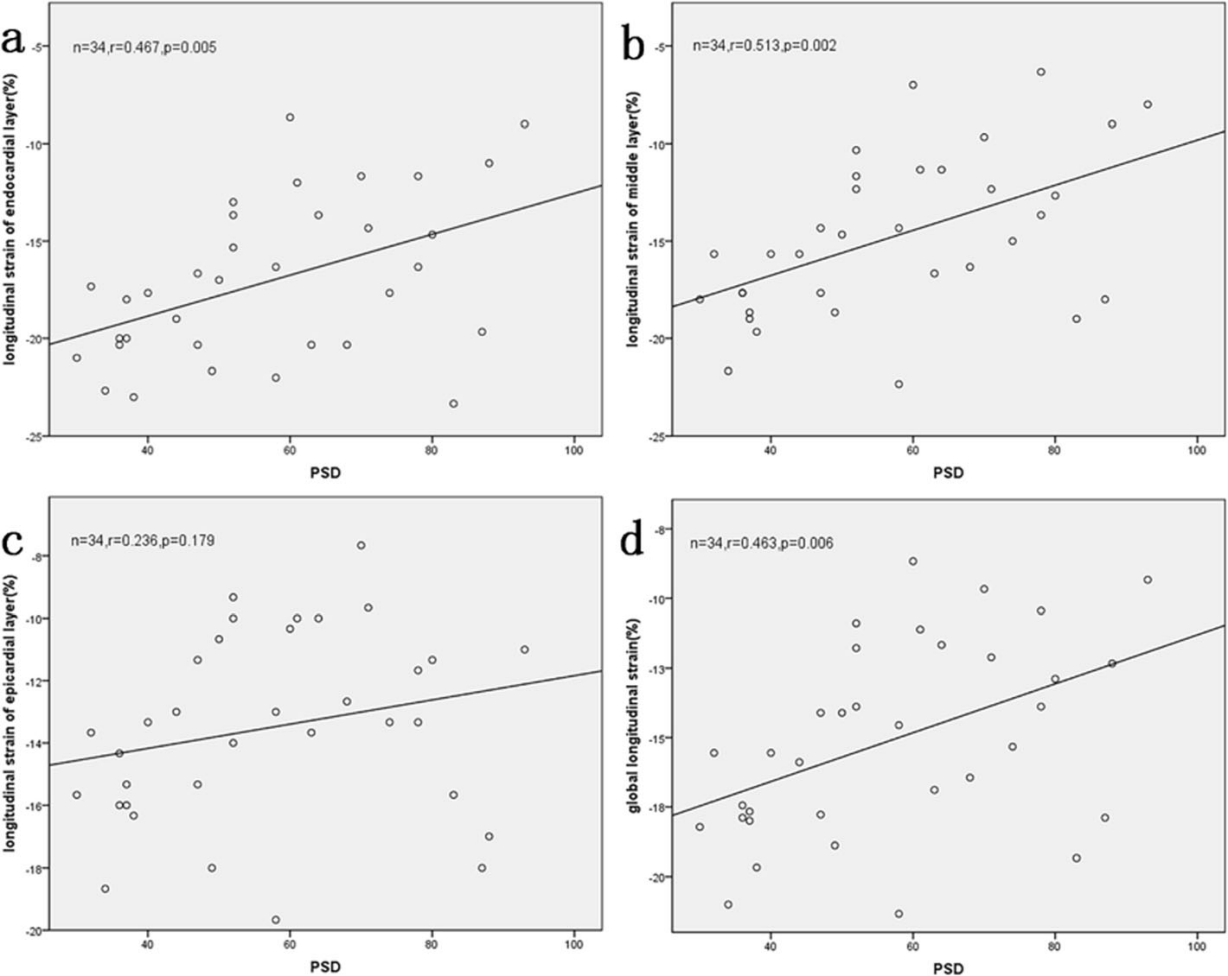
Correlation between PSD and peak LS of the sub-endocardial (**a**), mid-myocardial (**b**) and sub-epicardial layers (**c**), and the GLS (**d**) in MHD patients

We next examined the correlation between PSD and GLS with three-layer LV myocardial LS. As shown in Table 8 and Fig. 4, PSD was positively correlated with sub-endocardial and mid-myocardial LS and GLS (sub-endocardial, r = 0.467, *P* = 0.005; mid-myocardial, r = 0.513, *P* = 0.002; GLS, r = 0.463, *P* = 0.006). There was no significant correlation between PSD and sub-epicardial LS (*P* = 0.179).

**Table 8:** Correlation between PSD and LV three-layer myocardial LS and GLS

	Longitudinal strain (%)			
	Endocardial	Middle	Epicardial	Global
r-value	0.467	0.513	0.236	0.463
p-value	**0.005**	**0.002**	0.179	**0.006**

Bold number: *P* < 0.05

### ROC curve analysis of the accuracy of three-layer LV myocardial LS and CS in MHD patients

**Fig. 5:**
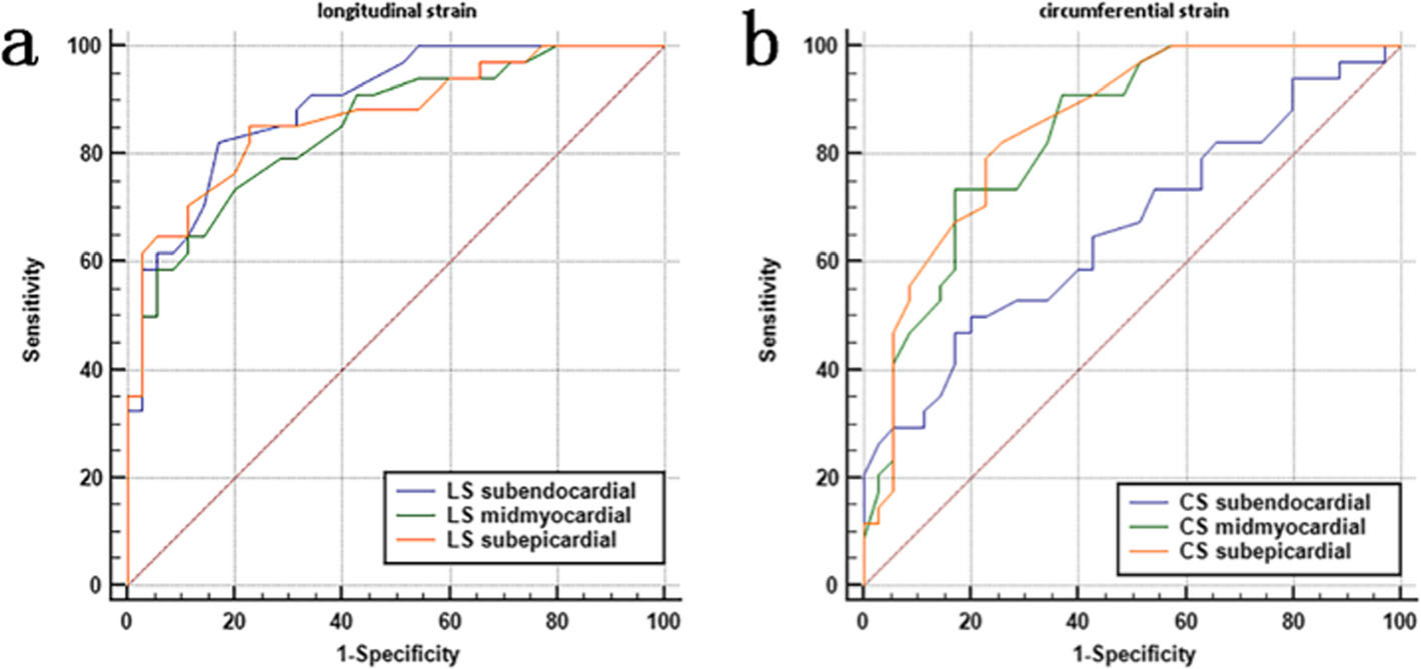
ROC curve analysis for evaluating the sensitivity and specificity of the peak LS (**a**) and peak CS (**b**) of different myocardial layers in identifying LV dysfunction in MHD patients

The AUC was analyzed to obtain the efficacy of three-layer LV myocardial LS and CS in the diagnosis of LV systolic dysfunction in MHD patients. The AUC values of sub-endocardial, mid-myocardial, and sub-epicardial LS myocardium in MHD patients were approximately 0.894, 0.852, and 0.870, respectively, and the cutoff values ​​were approximately − 21.15, − 18.33%, and − 17.08%, respectively. The sensitivity of sub-endocardial LS (85.3%) was higher than that of sub-epicardial (82.4%) and mid-myocardial LS (73.5%). The specificity of LS of the sub-endocardial LS (82.9%) was also higher than that of the mid-myocardial (80.0%) and sub-epicardial LS (77.1%). The AUC values of sub-epicardial, mid-myocardial, and sub-endocardial CS in MHD patients were approximately 0.852, 0.837, and 0.669, respectively, and the cutoff values ​​were − 11.65, − 17.86%, and − 24.33%, respectively. The sensitivity of sub-epicardial CS (82.4%) was higher than that of mid-myocardial (73.5%) and sub-endocardial CS (50.0%). However, the specificity of mid-myocardial CS (82.9%) was higher than that of sub-endocardial (80.0%) and sub-epicardial CS (74.3%) (Table 9, Fig. 5). Collectively, our findings indicate that sub-endocardial LS is an appropriate indicator of early stage cardiac dysfunction.

**Table 9:** ROC curve analysis of accuracy of LV three-layer myocardial LS and CS in MHD patients

Parameters	AUC	95%CI	Cut-off Value (%)	Sensitivity (%)	Specificity(%)	Youden index
LS sub-endocardial	0.894	0.796–0.955	−21.15	85.3	82.9	0.6521
LS mid-myocardial	0.852	0.746–0.926	−18.33	73.5	80.0	0.5353
LS sub-epicardial	0.870	0.767–0.939	−17.08	82.4	77.1	0.6244
CS sub-endocardial	0.669	0.545–0.778	−24.33	50.0	80.0	0.3000
CS mid-myocardial	0.837	0.728–0.915	−17.86	73.5	82.9	0.5639
CS sub-epicardial	0.852	0.746–0.926	−11.65	82.4	74.3	0.5664

AUC: area under the curve; 95% CI: 95% confidence interval; LS: longitudinal strain; CS: circumferential strain

### Intra-observer and inter-observer variability

We examined the intra-observer and inter-observer variability in our analysis. Twenty participants were randomly selected from the control and MHD groups. All observers were blinded to the subject type and measured the reproducibility of the three-layer LV myocardial LS, CS, GLS, GCS, and PSD. As shown in Table 10, our results suggest that our study generated reliable and consistent observations.

**Table 10:** Evaluation of intra-observer and inter-observer variability

	Intra-observer		Inter-observer	
	ICC	95%CI	ICC	95%CI
LS sub-endocardial	0.90	0.76–0.96	0.87	0.69–0.94
LS mid-myocardial	0.89	0.76–0.96	0.79	0.55–0.91
LS sub-epicardial	0.87	0.70–0.95	0.88	0.72–0.95
GLS	0.89	0.75–0.96	0.87	0.71–0.95
CS sub-endocardial	0.89	0.74–0.95	0.87	0.71–0.93
CS mid-myocardial	0.77	0.51–0.90	0.82	0.60–0.92
CS sub-epicardial	0.86	0.69–0.94	0.80	0.57–0.92
GCS	0.84	0.63–0.93	0.83	0.62–0.93
PSD	0.96	0.90–0.98	0.95	0.88–0.98

*ICC* intra-class correlation coefficients; 95% CI:95% confidence interval; *LS* longitudinal strain; *GLS* global longitudinal strain; *CS* circumferential strain; *GCS* global circumferential strain; *PSD* peak strain dispersion

## Discussion

Although MHD is the primary treatment for ESRD patients who are not candidates for kidney replacement, the mortality rate of MHD patients remains high and cardiovascular complications, such as heart failure and coronary heart disease, are the main factors associated with the high mortality of MHD patients [18]. Therefore, early detection of cardiac dysfunction in MHD patients has important clinical applications. In the present study, we used LST to evaluate the LS and CS of different LV myocardial layers of control and MHD patients and found that, despite the comparable LVEF between these two groups, MHD patients had altered LS and CS at different myocardial layers, higher TTP of 17 LV segments, and higher PSD compared to control subjects. Our findings suggest that LST holds value for early identifying LV dysfunction of MHD patients.

A previous study showed that hemodialysis did not significantly improve LV remodeling and systolic dysfunction in patients [19]. Consistent with that report, we first employed traditional echocardiography to assess the cardiac function of control subjects and MHD patients. We found that controls and MHD patients had a relatively comparable LVEF; however, MHD patients had significantly increased cardiac functional indexes, including LAD, LVIDD, LVIDS, IVSD, LVPWD, and LVMI. These findings suggest that ESRD patients exhibit LV remodeling with potential LV systolic dysfunction even after MHD treatment. Indeed, a previous study showed that ESRD patients on MHD had extensive pre- and post-loading factors such as sodium and water retention, anemia, malnutrition, valvular insufficiency, and hypertension [20]. The presence of a long-term arteriovenous fistula increased the pre-cardiac load in MHD patients [21]. To overcome increases in pre- and post-load, the LV develops physiological cardiac hypertrophy at an early stage to maintain normal cardiac output in MHD patients [22, 23].

Early studies have shown that the overall LS obtained by 2D-STE has become an objective and sensitive indicator for quantitative analysis of small changes in LV systolic function [24], which can be used to detect changes in regional myocardial blood supply earlier than LVEF [25]. LST based on 2D-STE technology is a new modality for evaluating wall motion. Animal experiments showed that LST can better assess the degree of myocardial infarction-induced damage at an early stage [26]. Previous studies showed that the mid- and epi- myocardia of LV are sensitive to post-load changes [27–29], and that the endocardial myocardium is sensitive to changes in volumetric load [27, 30], indicating that different LV myocardial layers exhibit different responses to myocardial insults. Thus, measuring layer-specific strain could be helpful to accurately assess the subtle changes in LV function during the progression of cardiac diseases. The present study used the layer-specific strain by 2D speckle tracking to appraise cardiac strain parameters of MHD patients, and revealed that MHD patients had significantly decreased myocardial LS of all layers compared to the control group. The impaired myocardial LS of all layers could be attributed to endothelial dysfunction and vascular injury [15], and to the observation that ESRD patients receiving MHD commonly present with anemia, secondary hyperparathyroidism, and arteriovenous fistula, all of which increase volume load and result in LV remodeling, hypertrophy, and fibrosis [31]. These pathological changes aggravate the progression of atherosclerosis and further heighten LV wall stress and stiffness [32], eventually leading to decreased endocardial LS [33]. As the disease progresses and dialysis time increases, myocardial fibrosis and LV hypertrophy is exacerbated, leading to a decrease in myocardial strain of each layer.

The cardiac myocardium can be divided into three layers: endocardial, middle, and epicardial. Approximately 75% of the endocardial and epicardial layers consist of longitudinal myocardium, while 25% of the middle layer consists of ring-shaped myocardium [34]. During myocardial contraction, the middle annular myocardium produces motion in the short axis direction. Compared with the control group, we found that MHD patients had comparable CS at the apical and the middle endocardial segments, indicating that LS was first impaired in MHD patients, while CS was only partially impaired at the early stage of cardiac impairment. Moreover, impaired CS was mainly observed in the middle and epicardial myocardium, which may be related to the fact that the middle myocardium has more ring-shaped myocardium. Since hemodialysis removes excess fluid from the body, the heart consequently increases LV contraction to maintain normal cardiac output and blood pressure [35]. However, MHD patients often have compromised cardiac function, and present with fluctuating blood pressure after hemodialysis. Since the mid- and epi-myocardium is sensitive to afterload [27–29], a large range of blood pressure fluctuation may underlie reduced CS in the mid- and epi-cardial myocardium of MHD patients.

LV TTP is the time from the R-wave of the electrocardiogram to the longitudinal peak strain of the LV 17 segments, and PSD measures the dispersion of the TTP of the LV 17 segments. Both TTP and PSD can be used to evaluate LV myocardial synchrony [36]. Compared with the control group, MHD patients had significantly delayed TTP and increased PSD, indicating decreased LV myocardial synchrony in MHD patients. This decreased LV synchrony could be attributed to increased sodium retention and high blood pressure. Indeed, some studies have shown that hypertension causes LV remodeling and alters myocardial electrophysiological properties, such as increased cardiomyocyte autorhythmicity and potential instability, myocardial electrophysiological conduction block, and myocardial excitation-contraction coupling dysregulation, all of which may lead to impaired LV systolic synchrony [37, 38]. Due to excessive volume overload, elevated peripheral blood pressure, and lack of ATP, LV contraction time is extended as a compensatory mechanism to insure adequate cardiac output [39]. Indeed, we found that PSD was positively correlated with GLS, which also indicates that the decrease in LV contraction in MHD patients is accompanied by a decrease in contractile synchrony. We also found that PSD was positively correlated with sub-endocardial and mid-myocardial GLS, but not with sub-epicardial GLS. This finding was probably due to the distribution of His bundle, left bundle branch, and Purkinje fiber mainly in sub-endocardium and mid-myocardium, thus rendering these myocardia more susceptible to the above factors [40].

Previously, Shi et al. [41] studied LV strain of young and middle-aged patients undergoing peritoneal dialysis, and found that sub-endocardial GLS was more sensitive to blood perfusion than the epicardium. Sun et al. [42] used LST technology to study LV function in ESRD patients, and reported that the curvature radius of the annular myocardial fiber was smaller than the curvature radius of the longitudinal myocardial fiber. Moreover, they found that the tension of the circumferential myocardial fiber was lower when deformed. Thus, they concluded that the LS of the myocardial fiber was more sensitive than CS. Leng et al. [43] also found that the decrease in LS occurred earlier than CS in response to external insults. In the present study, we analyzed the efficacy of three-layer strain parameters for predicting LV systolic function in MHD patients, and found that the AUC of the sub-endocardial LS was the largest with a cut-off value of − 21.15%, indicating that the sub-endocardial LS was the best index to evaluate LV systolic function. The AUC of the mid- and sub-epicardial CS was greater than that of the sub-endocardial CS. Also, the AUC of the sub-endocardial LS was greater than that of the mid- and epi-cardial CS. Thus, our findings are in line with the previous reports.

## Limitations

The limitations of this study should be acknowledged. First, our study was a single-centered study with a small sample size. Thus, we were not able to further divide MHD patients into subgroups. Second, the MHD patients in this study routinely took antihypertensive drugs, including calcium channel blockers and angiotensin converting enzyme inhibitors, and we did not rule out the possibility that the LV functional changes detected in MHD patients were already ameliorated by these treatments to some degree. Third, our study focused on LV longitudinal, circumferential strain, and synchrony, and did not include LV radial strain and torsion. Fourth, all MHD patients were assessed for coronary artery disease only by symptoms, electrocardiogram, myocardial enzyme profile, troponin, and echocardiography; they were not evaluated using angiography. Thus, our study did not rule out the possibility that some of these patients had subclinical coronary artery disease. Finally, the relationship between left ventricular load changes and left ventricular strain values revealed in MHD patients in this study needs to be further corroborated in the future.

## Conclusions

We report here that, although MHD patients had relatively normal LVEF, they had significantly reduced LS and CS of sub-endocardial, mid-myocardial, and sub-epicardial layers as detected by the LST. MHD patients also had significantly increased PSD, suggesting impaired LV systolic synchrony. Therefore, LST can detect changes of three-layer strain, LV systolic function, and dys-synchrony in MHD patients with high sensitivity. Our findings provide direct evidence that LST may be used clinically to detect cardiac dysfunction at an early stage in MHD patients to guide cardiac treatment.

## Data Availability

The datasets generated and/or analyzed during the current study are available in the Open Science Framework repository, https://mfr.osf.io/render?url=https://osf.io/bngwh/?action=download%26mode=render

## Abbreviations

2D-STE: Two-dimensional speckle tracking echocardiography
CS: Circumferential strain
ESRD: End stage renal disease
GCS: Global circumferential strain
GLS: Global longitudinal strain
IVST: Interventricular septal thickness at end-diastole
LAD: Left atrial diameter
LS: Longitudinal strain
LST: Layer-specific speckle tracking
LV: Left ventricular
LVEF: Left ventricular ejection fraction
LVIDD: Left ventricular internal diameter at end-diastole
LVIDS: Left ventricular internal diameter at end-systole
LVMI: Left ventricular mass index
LVPWT: Left ventricular posterior wall thickness at end-diastole
MHD: Maintenance hemodialysis
PSD: Peak strain dispersion
TTP: Time to peak longitudinal strain
